# Increase in Fentanyl-Related Overdose Deaths — Rhode Island, November 2013–March 2014

**Published:** 2014-06-20

**Authors:** Melissa C. Mercado-Crespo, Steven A. Sumner, M. Bridget Spelke, David E. Sugerman, Christina Stanley

**Affiliations:** 1 EIS officer, CDC; 2 Division of Unintentional Injury Prevention, National Center for Injury Prevention and Control, CDC; 3 Office of the State Medical Examiners, Rhode Island Department of Health

During November 2013–March 2014, twice as many all-intent drug overdose deaths were reported in Rhode Island as were reported during the same period in previous years. Most deaths were among injection-drug users, and a large percentage involved fentanyl, a synthetic opioid that is 50–100 times more potent than morphine (1). Clusters of fentanyl-related deaths have been reported recently in several states. From April 2005 to March 2007, time-limited active surveillance from CDC and the Drug Enforcement Administration identified 1,013 deaths caused by illicit fentanyl use in New Jersey; Maryland; Chicago, Illinois; Detroit, Michigan; and Philadelphia, Pennsylvania (2). Acetyl fentanyl, an illegally produced fentanyl analog, caused a cluster of overdose deaths in northern Rhode Island in 2013 (3).

The Rhode Island Department of Health (RIDOH) requested CDC’s assistance in describing and determining risk factors for recent fentanyl-related overdose death cases. CDC abstracted records from RIDOH’s Office of State Medical Examiners, Division of Vital Records, and Prescription Monitoring Program, with the assistance of local staff members. A fentanyl-related overdose death was defined as a death that occurred during November 2013–March 2014 in which fentanyl was listed as the official cause of death, a contributor to the cause of death, or in which toxicology reports identified fentanyl levels above the detection limit (≥2 ng/mL) by enzyme-linked immunosorbent assay.

Preliminary analyses show that fentanyl-related overdose deaths accounted for 52 (31.5%) of the 165 unintentional overdose deaths reported during November 2013–March 2014. Most decedents did not have active fentanyl prescriptions; the fentanyl appeared to originate from illicit sources and was not acetyl fentanyl–related. Although fentanyl-related overdose deaths were widespread in Rhode Island, most cases occurred in Providence and surrounding urban areas. CDC is currently conducting additional data analyses to determine whether the prescription monitoring program records or medical records of the decedents might help identify others at high risk for similar outcomes.

CDC collaborated with RIDOH to develop an emergency regulation that requires all Rhode Island emergency departments to report fatal and nonfatal opioid overdose cases within 48 hours to RIDOH. CDC recommended that RIDOH continue and expand its efforts to make naloxone, a prescription drug that helps reverse the effects of opioids, accessible for prior drug overdose patients and their families.
